# Nucleosome Organization around Pseudogenes in the Human Genome

**DOI:** 10.1155/2015/821596

**Published:** 2015-05-04

**Authors:** Guoqing Liu, Fen Feng, Xiujuan Zhao, Lu Cai

**Affiliations:** ^1^The Institute of Bioengineering and Technology, Inner Mongolia University of Science and Technology, Baotou 014010, China; ^2^School of Mathematics, Physics, and Biological Engineering, Inner Mongolia University of Science and Technology, Baotou 014010, China

## Abstract

Pseudogene, disabled copy of functional gene, plays a subtle role in gene expression and genome evolution. The first step in deciphering RNA-level regulation of pseudogenes is to understand their transcriptional activity. So far, there has been no report on possible roles of nucleosome organization in pseudogene transcription. In this paper, we investigated the effect of nucleosome positioning on pseudogene transcription. For transcribed pseudogenes, the experimental nucleosome occupancy shows a prominent depletion at the regions both upstream of pseudogene start positions and downstream of pseudogene end positions. Intriguingly, the same depletion is also observed for nontranscribed pseudogenes, which is unexpected since nucleosome depletion in those regions is thought to be unnecessary in light of the nontranscriptional property of those pseudogenes. The sequence-dependent prediction of nucleosome occupancy shows a consistent pattern with the experimental data-based analysis. Our results indicate that nucleosome positioning may play important roles in both the transcription initiation and termination of pseudogenes.

## 1. Introduction

Pseudogenes are produced from protein-coding genes during evolution. Though highly homologous with their parent genes, pseudogenes are unable to synthesize functional protein due to the defects in their sequences. There are two major types of pseudogenes: duplicated pseudogenes and processed pseudogenes (or retropseudogenes). The former type is created by genomic duplication and the latter by retrotransposition [1, 2]. For each type, the abnormalities occurred in either the protein-coding regions or the regulatory regions of parent genes leading to the loss of protein-coding ability of pseudogenes. Duplicated pseudogenes are often distributed in the flanking of the parent genes and may still maintain the upstream regulatory sequences of their parents due to their duplicative origin. Processed pseudogenes are usually characterized by absence of intron-like segments, decayed poly A tail, frame shifts, and premature stop codons. Processed pseudogenes are thought to be nonautonomous retrotransposons which are probably mobilized by long interspersed elements (LINEs), a kind of autonomous retrotransposons in the genome [3, 4]. Processed pseudogenes occur in a great number of eukaryotes, especially in mammalian genomes [5, 6].

Many unexpected discoveries of biological functions for pseudogenes challenge the popular belief that pseudogenes are nonfunctional and simply molecular fossils. A nitric oxide synthase (NOS) pseudogene functions as a regulator of the paralogous protein-coding neuronal nitric oxide synthase (nNOS) gene by producing antisense RNA that forms a duplex with some of the gene's mRNA [7, 8]. The Makorin1-p1 pseudogene in mouse regulates the stability of the mRNA of its homologous Makorin1 gene probably by producing RNA which competes for the freely available repressor molecules that inhibit the homologous gene expression [9]. Some pseudogenes can also compete with their parent genes for microRNA binding, thereby modulating the repression of the functional gene by its cognate miRNA [10]. The transcription of MYLKP1 pseudogene, which is upregulated in cancer cells, creates a noncoding RNA (ncRNA) that inhibits the mRNA expression of its parent MYLK gene [11]. Moreover, recent studies have documented that a subset of pseudogenes generates endogenous small interfering RNAs (endo-siRNAs) and suppresses gene expression by means of the RNA interference pathway in mouse oocytes [12, 13], subsequently in rice [14], most lately in African Trypanosoma brucei [15], a unicellular eukaryote. These observations suggested that pseudogenes might be an alternative source of natural antisense transcripts that regulate the activity of sense transcripts of their parent genes. Besides, pseudogenes may have a whole set of functions related to intracellular immunobiology [2, 16, 17].

The variety of known or suspected pseudogene functions discovered to date suggests that pseudogenes as a whole have a wide range of previously unsuspected functions. Of the functions, RNA-level functions are of great importance and are most frequently discussed. The prerequisite of understanding the RNA-level functions of pseudogenes is to explore their transcriptional activity. It has been shown that the nucleosome, a fundamental composing unit of the chromatin structure in eukaryotes, affects gene transcription in that it modulates the accessibility of underlying genomic sequence to proteins [18]. How does nucleosome positioning affect pseudogene transcription? Seeking to answer the question, we analyze the nucleosome organization around the pseudogenes in human. Nucleosome occupancy is measured by both a sequence-dependent computational model and experimental data [19]. The computational model emphasizes the sequence-dependency of nucleosome positioning, while the nucleosome occupancy inferred from in vivo experimental data reflects the joint effect of DNA sequence and other external factors, such as chromatin remodeler, DNA methylation, histone modification, and polymerase II binding, on nucleosome positioning [19–21]. The two methods may have different implications for the dependency of pseudogene transcription on chromatin structure.

## 2. Materials and Methods

### 2.1. Materials

#### 2.1.1. Transcribed and Nontranscribed Pseudogenes

A total of 201 consensus pseudogenes, including 124 processed pseudogenes and 77 duplicated pseudogenes, were identified in ENCODE regions [22]. Of the ENCODE pseudogenes, 38 pseudogenes have evidence of transcription, and others are considered to be nontranscribed. The sequences and annotation information (genomic position, strand, and positions of start positions and end positions) of the pseudogenes mapping to the human genome (hg18) were retrieved from UCSC (http://www.genome.ucsc.edu/). The type and transcriptional information of the pseudogenes were downloaded from the pseudogene database (http://www.pseudogene.org/). The number of transcribed pseudogenes in ENCODE regions is too small, so we refer to the genome-wide transcribed processed pseudogenes that were identified by Harrison et al. [23]. The annotation of the 192 transcribed processed pseudogenes that corresponds to the human genome (version hg18) was taken from the pseudogene database (http://www.pseudogene.org/). The transcribed processed pseudogenes were identified by mapping three sources of expressed sequences (Refseq mRNAs, Unigene consensuses, and ESTs from dbEST) onto the processed pseudogenes. Oligonucleotide microarray data was used to further verify the expression of the selected transcribed pseudogenes [23]. The sequences surrounding the start sites and end sites of the transcribed pseudogenes were retrieved from the human complete genome (hg18) by using the positional information of the pseudogenes. The statistics of the pseudogenes are listed in Table 1.

#### 2.1.2. Human Nucleosome Occupancy

Experimental data-based nucleosome occupancy profile mapping to the human genome (hg18) was taken from Schones et al. [19]. It was based on maps of nucleosome positions in both resting and activated human CD4+ T cells generated by direct sequencing of nucleosome ends using the Solexa high-throughput sequencing technique. The two nucleosome profiles (resting and activated) have a resolution of 10 bp. We applied cubic spline fitting to each of the profiles to obtain nucleosome occupancy at each genomic site. We also estimated nucleosome occupancy by a sequence-dependent computational model described in detail in the Methods section.

### 2.2. Methods

#### 2.2.1. Conformational Energy Calculation

Conformational energy is to be calculated on the basis of the geometrical description of DNA double helix structure. According to Cambridge Convention [24], each base pair of DNA is viewed as a rigid board, and its position relevant to its neighbor is specified by roll, tilt, twist, slide, shift, and rise. Nucleosomal DNA bending appeared to be due to periodic variations in both roll and tilt in the crystal structure 1kx5 [18]. The periodic changes reflected the helix twisting that altered the rotational position of each base-pair step (or dinucleotide step) relative to the dyad. In addition to the general trend of periodic changes, variations in the roll and tilt at each base-pair step were also dependent on the property of individual dinucleotide.

Nucleosomal DNA deformation is viewed as forced bending. It is assumed that torque *F*_*b*_ is uniformly distributed along the DNA. We consider DNA bending to be analogous to the bending of a rod of multiple segments with variable stiffness. For a bending force exerted by the histone octamer on a segment of the DNA, the conformational energy at each step along the sequence depends on both the corresponding dinucleotide flexibility and the phasing of the dinucleotide with respect to the dyad. According to simple elastic model, deformations of roll and tilt from their equilibrium values at dinucleotide step *i* are described as

(1) $$\begin{array}{c} \rho \left(i\right)-\rho_{0}\left(i\right)=\frac{F_{b}\cos\Omega_{i}}{k_{\rho }\left(i\right)},\\ \tau \left(i\right)-\tau_{0}\left(i\right)=\frac{F_{b}\sin\Omega_{i}}{k_{\tau }\left(i\right)}. \end{array}$$

The bending energy is then calculated by

(2) Ebi=12kρiρi−ρ0i2+12kτiτi−τ0i2=Fb22kρicos⁡2Ωi+Fb22kτisin2Ωi,

where *ρ*(*i*) and *τ*(*i*) are, respectively, the actual roll and tilt angle at dinucleotide step *i*, *ρ*_0_(*i*) and *τ*_0_(*i*), which are dependent on the dinucleotide at step *i*, are, respectively, the roll and tilt without torque, *k*_*ρ*_(*i*) and *k*_*τ*_(*i*) are the dinucleotide-dependent force constants, and *Ω*_*i*_ is the accumulated twist (*ω*) at the center of step *i*, counted from the dyad position. For 147 bp nucleosomal core DNA, its structure is symmetrical with respect to the dyad that is located at the centeral nucleotide, and the dinucleotide steps from the dyad are labeled as *i* = ±1, ±2, ±3,…, ±73 towards downstream and upstream directions. The step ±1 is half step away from the dyad; thus the accumulated twist is calculated as follows:

(3) $$\begin{array}{c} \Omega_{i}=\left\{\begin{array}{cc} 0.5\omega_{1}+\overset{i}{\underset{2}{\sum }}\omega_{i}, & \text{if}\;\;i>0,\\ -\left(0.5\omega_{-1}+\overset{-2}{\underset{i}{\sum }}\omega_{i}\right), & \text{if}\;\;i<0. \end{array}\right. \end{array}$$

The bending energy for the central *L*-bp segment of a nucleosomal DNA is the sum of corresponding dinucleotide steps:

(4) Eb=∑−L−1/2L−1/2Ebi=∑−L−1/2L−1/2Fb22kρicos⁡2Ωi+Fb22kτisin2Ωi,

where *L* is a positive odd number and less than or equal to 147.

In (4), *F*_*b*_ is determined by utilizing its relationship with the total bending angle of the core DNA. In the crystal structure of core particles, about 10 bp at each end has no contact with the histone octamers, and therefore the sequence dependency of nucleosome positioning is reflected merely in the central 129 bp part of the nucleosomal DNA. The central 129 bp part of the nucleosomal core DNA bends around histone octamer about 579° (*α*) under the stress of *F*_*b*_, and the *α* is due to contribution of *ρ* and *τ* at every step:

(5) $$\begin{array}{c} \alpha =\overset{64}{\underset{i=-64}{\sum }}\left[\rho \left(i\right)\cos\Omega_{i}+\tau \left(i\right)\sin\Omega_{i}\right]. \end{array}$$

Combining (1) and (5) leads to

(6) $$\begin{array}{c} F_{b}^{}=\frac{\alpha -\sum_{i}^{}\rho_{0}\left(i\right)\cos\Omega_{i}-\sum_{i}^{}\tau_{0}\left(i\right)\sin\Omega_{i}}{\sum_{i}^{}\left(\cos^{2}\Omega_{i}/k_{\rho }\left(i\right)\right)+\sum_{i}^{}\left(\sin^{2}\Omega_{i}/k_{\tau }\left(i\right)\right)}. \end{array}$$

The empirical parameters of our model for conformational energy calculation consist of force constants (*k*_*ρ*_ and *k*_*τ*_) and roll and tilt angles (*ρ*_0_ and *τ*_0_) for 10 dinucleotides at the equilibrium state (Table 2). The dinucleotide-dependent parameters *ρ*_0_ and *τ*_0_ averaged over a large pool of DNA-protein complexes and force constants *k*_*ρ*_ and *k*_*τ*_ are taken from the paper of Morozov et al. [25]. A constant *ω* = 34.8°, average twist for the 1kx5 X-ray crystal structure of nucleosome-bound DNA, was used for all dinucleotide steps.

#### 2.2.2. Nucleosome Occupancy Estimation

According to Boltzmann distribution, the potential of forming a nucleosome which centers at position *j* in a DNA segment of *N* bp is defined as

(7) $$\begin{array}{c} S_{j}=e^{-\beta E_{j}}, \end{array}$$

where *β* = 1/*k*_*B*_ *T*, *k*_*B*_ is Boltzmann constant, *T* is the room temperature, *M* = 147 (nucleosome size), and *E*_*j*_ is the deformation energy of the underlying DNA of the nucleosome which occupies positions *j* − (*M* − 1)/2 through *j* + (*M* − 1)/2. For simplicity, we assume *β* = 1 in calculation. Nucleosome occupancy at the base-pair position *j* is measured by the average of the nucleosome formation potentials over *l*-bp window:

(8) $$\begin{array}{c} O_{j}=\frac{\sum_{i=j-\left(l-1\right)/2}^{j+\left(l-1\right)/2}S_{i}}{l}. \end{array}$$

In this study, *l* = 51, of which performance was validated in our other study (unpublished).

Normalized nucleosome occupancy at every base-pair is calculated by the log-ratio between the corresponding absolute nucleosome occupancy *O*_*i*_ and the average nucleosome occupancy 〈*O*_*i*_〉 per base-pair across the genome as

(9) $$\begin{array}{c} O_{j}^{\text{nor}}=\log\frac{O_{j}}{\langle O_{j}\rangle }. \end{array}$$

## 3. Results and Discussion

### 3.1. Experimental Nucleosome Occupancy around Pseudogenes

**Figure 1:**
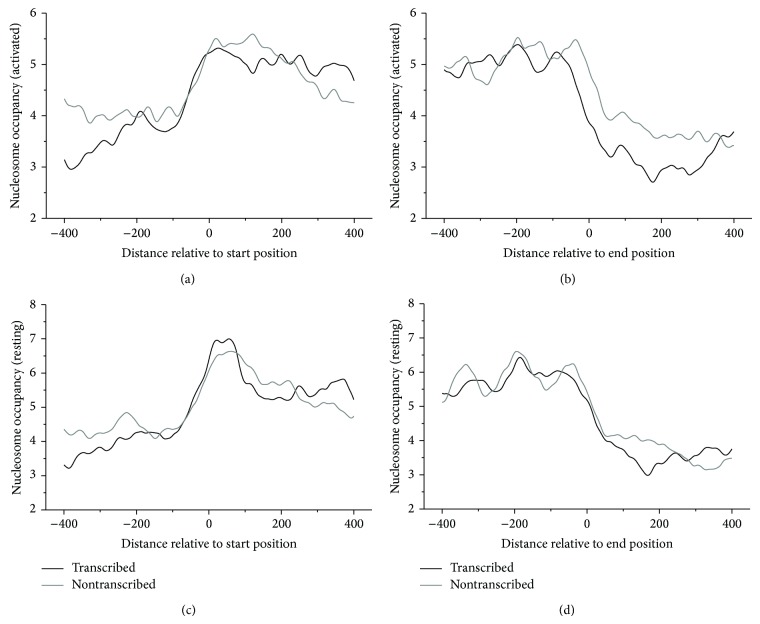
Experimental nucleosome occupancy around start positions and end positions of pseudogenes.

As shown in Figure 1, nucleosome occupancy exhibits clear distribution pattern around the start positions and end positions of pseudogenes: (1) nucleosomes are depleted upstream and enriched downstream of the start positions; (2) nucleosomes are enriched upstream and depleted downstream of the end positions; (3) the nucleosome depletion pattern is similar between transcribed pseudogenes and nontranscribed pseudogenes; (4) nucleosome occupancy profile shows similar pattern between resting and activated human CD4+ T cells.

An obvious nucleosome depletion detected upstream of the start positions of transcribed pseudogenes, suggesting that the nucleosome depletion at the region may promote the pseudogene transcription by exposing the underlying sequence in a linker region, which is accessible for transcription factor binding. A similar depletion at the region downstream of the end positions of transcribed pseudogenes might imply the role of nucleosome positioning in transcription termination by facilitating the sequence to form hairpin structure to terminate transcription. Note that the nucleosome depleted regions detected upstream of the start positions and downstream of the end positions of transcribed pseudogenes match well with the transcription start region and transcription end region of the pseudogenes, respectively.

As compared with transcribed pseudogenes, nucleosome depletion both upstream and downstream of the nontranscribed pseudogenes is unexpected since nucleosome depletion in those regions is thought to be unnecessary in light of the nontranscriptional property of those pseudogenes.

### 3.2. Sequence-Dependent Prediction for Nucleosome Occupancy around Pseudogenes

**Figure 2:**
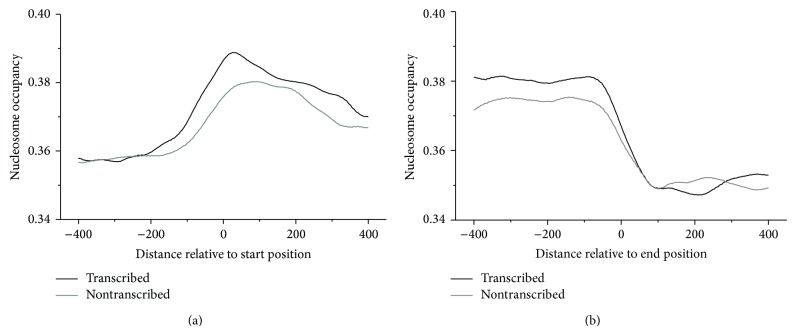
Calculated nucleosome occupancy around start positions and end positions of pseudogenes. Analysis of variance (ANOVA) shows significant differences of average nucleosome occupancy between transcribed and nontranscribed pseudogenes (*P* < 0.001).

The overall distribution trend of experimentally determined nucleosome occupancy around both start positions and end positions of pseudogenes is reproduced successfully by our computational model (Figure 2). It has been demonstrated in the previous study that predicted occupancy has a better correlation with in vitro nucleosome occupancy than in vivo occupancy [26], as our prediction depends solely on the physical properties of DNA and reflects the sequence-dependent nucleosome-forming ability. In the present paper, the depletion of nucleosomes both upstream of start positions and downstream of end positions and enrichment of nucleosomes both downstream of start positions and upstream of the end positions merely reflect the sequence properties to form nucleosome. The consistence of the overall distribution trend of nucleosome occupancy around pseudogenes between sequence-dependent prediction (Figure 2) and in vivo case (Figure 1) suggests that the DNA sequence is an important determinant of nucleosome positioning in human as in yeast. Our sequence-based model predicted nucleosome depletions both upstream and downstream of the nontranscribed pseudogenes. This suggests that the in vivo nucleosome depletions surrounding the nontranscribed pseudogenes are dominated by DNA sequence.

### 3.3. The Effect of Sequence Degeneration of Pseudogenes on Nucleosome Formation

Pseudogenes provide a natural resource of relics for researchers to explore the chromatin response to sequence mutations that are enriched in pseudogenes. Specifically, a number of structurally similar but not identical pseudogenes can be produced from a single functional gene during evolution. In particular, each of the high-transcriptional ribosomal protein genes tends to have many, in some cases over 100, pseudogenes. A simple way to test the possible change of nucleosome distribution over pseudogenes is to correlate the nucleosome occupancy over the pseudogenes with their evolutionary distances. To do this, we first downloaded the annotation (hg16-based) for 2536 ribosomal protein (RP) pseudogenes [27] from the pseudogene database (http://pseudogene.org/) and then remapped them onto the hg18 human genome using Lift program accessed at http://www.genome.ucsc.edu/. 2401 RP pseudogenes were successfully mapped. From them, duplicated pseudogenes and pseudogenic fragments that account only a small percentage of pseudogenes were removed. Finally, we retained 1931 processed pseudogenes whose sequences and annotations (GC content, DNA identity to their ancestral genes, etc.) are available at http://pseudogene.org/. We computed the average nucleosome occupancy over each pseudogene from the hg18-based experimental nucleosome reads data (the same to the procedure described in Section 2.1.2). Sequence-dependent predictive model was also applied to the pseudogenes to get average nucleosome occupancy over each one. The correlations among the variables for each RP pseudogene family were computed (Table 3).

Our data clearly illustrate that predicted nucleosome occupancy over pseudogenes tends to positively correlate with their DNA identity, suggesting that the ability of the pseudogenes to form nucleosome(s) tends to decline in the process of their evolution. However, we did not detect a positive correlation between experimental nucleosome occupancy and DNA identity. There are three possible reasons for this. Firstly, the effects of some nonsequence factors which are likely to play a larger role in nucleosome positioning in human than in simple eukaryotes, such as yeast, exceed the sequence-induced effect on nucleosome positioning [19]. Secondly, it is also possible that the mutations occurring in some pseudogenes are so little and trivial that they cannot bring about a significant effect on the nucleosome-forming ability of pseudogenes. Thirdly, the high substitution rates in nucleosome-enriched regions [28] are likely to result in the weak negative correlation between nucleosome occupancy and pseudogene identity.

We also found a significant correlation between pseudogenes' divergence and their predicted nucleosome occupancy, indicating again the decreasing trend of nucleosome-forming ability of pseudogenes during their degradation process. Furthermore, there is a strong positive correlation of predicted nucleosome occupancy of pseudogenes with their GC content, consistent with the previous finding that GC content dominates intrinsic nucleosome occupancy [29]. The GC-dependency of nucleosome occupancy and the decrease of GC content of pseudogenes with time [6] could explain the reduced intrinsic preference of pseudogenes for nucleosome-forming during evolution.

## 4. Conclusion

In this report, we analyzed the organization of nucleosomes around pseudogenes and compared between transcribed and nontranscribed pseudogenes. Experimental data-based analysis shows nucleosome depletion both upstream of the start positions and downstream of the end positions of transcribed pseudogenes, suggesting that nucleosome positioning plays an important role in both transcription initiation and transcription termination of pseudogenes. A similar depletion of nucleosomes is detected for nontranscribed pseudogenes, which is likely to be caused by sequence-dependent nucleosome-inhibitory effect. We also applied a sequence-dependent model for calculating nucleosome occupancy to pseudogenes and obtained consistent pattern with experimental nucleosome organization.

## Figures and Tables

**Table 1:** The statistics of pseudogenes.

	Transcribed	Nontranscribed
Processed	192	106
Duplicated	0	57

Total	192	163

**Table 2:** The dinucleotide-dependent force constants and parameters *ρ*_0_ and *τ*_0_.

Step	*k*_*ρ*_	*k*_*τ*_	*ρ*_0_	*τ*_0_
AA/TT	0.2	0.406	0.76	−1.84
AT	0.124	0.641	−1.39	0
AG/CT	0.077	0.28	3.15	−1.48
AC/GT	0.085	0.302	0.91	−0.64
TA	0.064	0.365	5.25	0
TG/CA	0.059	0.393	5.95	−0.05
TC/GA	0.097	0.408	3.87	−1.52
GG/CC	0.075	0.218	3.86	0.4
GC	0.057	0.256	0.67	0
CG	0.04	0.255	4.25	0

**Table 3:** The proportion of significant Spearman correlations between nucleosome occupancy and pseudogene characteristics with regard to 79 RP pseudogene families.

	pgene GC	Identity^a^	Divergence^a^
Predicted	68/77^b^ (*R* = 0.817, 68+)^c^	41/77 (*R* = 0.589, 39+)	41/77 (*R* = −0.622, 2+)
Experimental	3/77 (*R* = −0.02, 1+)	3/77 (*R* = −0.106, 1+)	5/77 (*R* = 0.026, 2+)

^a^The “Identity” and “Divergence” of pseudogenes from the coding sequences of their functional RP genes were taken from Zhang et al. 2002 [27]. The “Divergence” was computed with the program MEGA2, using the Kimura two-parameter model and pairwise deletion.

^b^Among 79 RP pseudogene families, there are two RP pseudogene families whose lengths are not up to 129 bp, a minimum required size for nucleosome occupancy prediction.

^c^The average of the significant Spearman correlation coefficients and the number of positive significant correlations were indicated in the parenthesis.
